# The Role of Zinc in the T-Cell Metabolism in Infection Requires Further Investigation - An Opinion

**DOI:** 10.3389/fimmu.2022.865504

**Published:** 2022-03-10

**Authors:** Consolato M. Sergi

**Affiliations:** Anatomic Pathology Division, Children’s Hospital of Eastern Ontario (CHEO), University of Ottawa, ON, Ottawa, Canada

**Keywords:** zinc, supplementation, T-cell, metabolism, thymus

Thymus depletion is a usual finding in human-immunodeficiency virus (HIV) infected patients, but a refurbishment of the thymus function in children has been proposed in some virologic responders treated with multidrug combinations of antiretroviral agents (1–3). In addition, studies from children with acquired immunodeficiency syndrome (AIDS) have clarified the pathogenesis of some primary tissue lesions (2, 4, 5). These studies are highly relevant to understanding the direct and indirect sequels associated with HIV infection.

In the last two decades, we have observed a thrilling interest in pursuing the topic of the interaction between HIV and hematopoietic stem cells of progenitor origin (HSPCs, hematopoietic stem progenitor cells) (6). Despite the great interest and research, numerous aspects are still unclear. It remains ambiguous whether the hematopoietic stem cells of progenitor origin can behave as virologic reservoirs. In fact, it is known that some investigations have suggested that the latently infected stem cells are fundamental in the bone marrow of patients infected with HIV or suffering from AIDS. However, other authors have promoted negative results that have challenged such data’s reproducibility. Facing this dilemma, it is probably compulsory to start elucidating this controversy. Suppose an arm of HSPC studies of HIV infection entails dynamics investigations in the stages (both early and late) of the disease to recognize the influence on the pathogenesis of AIDS. In that case, reduced quantities and functional damage of multipotent cells with myeloid and lymphoid progenitor ancestry in HIV infection may promote indicators in hematology. Therefore, trace elements and supplementations have been targeted to exponentially boost the immunologic function of both healthy individuals and patients with different diseases.

Zinc (Zn) is an indispensable micronutrient with a re-discovered interest in nutritional medicine and supplementation (7). Zn is key to almost every aspect of the evolution of our human health. Zn, a transition metal, is grouped with cadmium and mercury in the periodic table. Zn is second only to iron as the most abundant trace mineral in humans in our body. Research has focused on the utility of this transition metal in the immunologic system (7–10). It seems that this trace element may boost immune function, soothe glycemia levels, and help keep cardiac, ocular, and cutaneous systems healthy, targeting the T-cell metabolism intimately (11–13).

T -cell receptor-derived excision circles (TRECs) are DNA portions in small circles generated in T-lymphocytes in a specific time. The period is their transition through the thymus as the T cells rearrange their T-cell receptor (TCR) genes (14–16). Thus, their occurrence implies the maturation of T lymphocytes. TRECs are reduced in severe combined immunodeficiency disease (SCID) and in other conditions associated with immunodeficiency, such as the Chromosome 22q11.2 Deletion (DiGeorge Syndrome) (15, 17–22). Recently, Iovino et al. (23) stated clearly that Zn supplementation after HSCT causes an increase in TRECs and CD4+ naïve lymphocytes. It also prevented the reactivation of the Torque Teno Virus (TTV), a universal virus with identified in more than half of the general human population substantially worldwide. It is considered a blood-borne virus with parenteral, sexual, mother-to-child, and other ways of transmission (24). Despite being proposed to be an etiologic agent of several non-oncological diseases (e.g., liver diseases, respiratory tract diseases, AIDS) and cancer, there has been no or poor support until now. Still, there is some evidence that TTV may play a role in developing autoimmune reactions (24).

The versatility of the thymus to generate naive T-lymphocytes exhibiting a vast range of T-cell receptor mixture is an essential premise to developing an efficient immune system able to recognize an extensive range of antigens throughout life. Naïve T cells need to be activated and expanded to generate memory T cells establishing the peripheral T cell repertoire by migrating from the thymic microenvironment to peripheral lymphoid tissue. Although the thymus, which is the central organ of the human body able to target the T lymphopoiesis, is highly susceptible to many insults, it also has an extraordinary capacity to regenerate after damage. Yet, the prolonged lymphopenia and delayed thymic reconstitution following HSCT negatively affect clinical outcomes, especially in the elderly. In this age group, the thymic gland has already undertaken size reduction, which is physiological in nature, decreasing its ability to generate new T-lymphocytes (3).

In a pilot study on Multiple Myeloma (MM) patients, who were eligible to auto-HSCT, Iovino et al. performed a preliminary or pilot investigation. They studied nine patients per group with or without Zn supplementation finalized the study to assess the tolerability of the therapy, the rate of adverse events (AE), and the sequels on the substitute immunological consequences. After HSCT, five patients initially had AEs judged to be caused by zinc supplementation. *A posteriori*, it has been identified that these AE may have been probably multifactorial. No severe AEs were reported. With regard to the immunological factors, no statistical differences were detected between the two groups at the time of the registration. An exception was noted with regard to the higher level at baseline of CD8+ central memory (CD8 + CM) (circulating CD8+ cells) in favor of the control group. The two groupings also did not differentiate in TRECs at baseline, but, remarkably, the control group showed higher values at the time-point before HSCT. There was no difference in TRECs in absolute number between the two groupings at days +30 and +100. TTV load remained low in both groups from baseline until day +30, but at day +100, the control group showed TTV replication higher levels of CD4 + CD45RA+ terminal effector (CD4 + TE), and a higher level of circulating CD4+ central memory (CD4 + CM). The data on the circular, single-stranded DNA TTV may be significant and harbinger of more studies in the future. The median average of circulating CD4+ T-lymphocytes (naïve) demonstrated no differences between the two groupings across timelines. However, CD4+ naïve lymphocytes and TRECs remarkably increased from day +30 through +100 in the Zn group only. The relative boost of TRECs concerning pre-HSCT levels reached a value of 6.1-fold in the Zn group, which is remarkably higher equated to the value of a 1.8-fold rise in the control group.

Recently, we identified some mechanisms underlying germline variations impairing the domain member 11 of the caspase recruitment 11 (CARD11)-B cell leukemia of chronic lymphocytic type 10 (BCL10)-mucosa-associated lymphatic tissue 1 (MALT1) or CBM complex (25). This complex is intimately connected with various human diseases, including SCID, allergy/atopy, and lymphoproliferative disorders. The CBM signalosome complex operates as a molecular “bridge” between antigen receptor signaling on the cell surface and the triggering of the nuclear factor-kappa B (NF-κB), a protein complex regulating DNA transcription, cytokine generation, and overall cell survival. In addition, there is the triggering of two signaling axes, i.e., JNK or c-Jun N-terminal kinases, and mTORC1 or mammalian target of rapamycin complex 1. JNKs are a group of protein kinases that exhibit a central role in stress signaling pathways NF-κB and Activator Protein 1 (AP-1) transcriptional factors that regulate the expression of numerous genes involved in angiogenesis, autochthonous tumor growth, and tumor metastasis. Treatment of cancer cell lines with zinc reduces the expression of growth factors, interleukins, and matrix proteins. Thus, Zn reduces the expression of ICAM-1 or, also known as, intercellular adhesion molecule of type 1. In addition, Zn represses tumor cell invasiveness and, substantially, the cellular adhesion (26). Also, Zn deficiency-triggered stress of oxidative type could impact cell signaling, including mainly transcription factors containing Zn finger motifs. In addition, Zn may also act on other oxidant-sensitive transcription factors such as the NF-κB mentioned above and AP-1. An impairment of the nuclear transport of the active transcription factor may lead to a small amount of NF-κB-dependent genes being expressed that could be favorably involved in various steps of Zn deficiency-associated pathology, underlining the prominent role of Zn in this process, which seems to be paramount and critical in senility and in human conditions and syndromes associated with an accelerated process of aging featuring changes in biological, physiological, and behavioral processes (27).

Zinc deficiency is not a trivial absence of a chemical element. Clinically, it causes depression of the immune system and progressive and accelerated atrophy of the thymus (28). In a consequence of it, there is a decrease in circulating recent thymic emigrants (RTE). It is true that no statistical differences were found in baseline serum levels of zinc. Nevertheless, Iovino et al.’s investigations are praiseworthy. Although they pointed out that their Droplet Digital PCR based study should be considered a pilot investigation only and a larger number of patients should be enrolled in randomized clinical trials with an associated animal model for mechanistic insights are necessary, it is worthy that the supplementation of this element can be attempted to try to promote the thymic regeneration after damage. There was a significant increase of CD4+ naïve cells and TRECs from day +30 until day +100 only in the zinc-treated group. TRECs are the most accurate measurement for evaluating the functionality of the thymus and the production of RTEs in practice (29). Therefore, there is substantial some good optimism because the CD4+ naïve cells and TRECs at day +100 clearly indicate an augmentation of the production of RTEs triggered by zinc supplementation. Substantially, higher levels of RTEs after allogeneic HSCT are correlated with an improvement in the prognosis of these patients, who experience a decreased risk of infection and a relatively lower ratio of onset of graft versus host disease (GvHD) (30–32).

In our opinion, there is some evidence that Zn may be of benefit to patients receiving HSCT and in individuals affected with other immunologic deficiencies, such as CARD11 deficiency (25, 33). However, randomized clinical trials with numerous patients and animal models for mechanistic data are warranted. It is crucial to set up such trials in the future, starting multinational cooperation and liaising effectively with experts in veterinary pathology. Overall, there is a reasonable ground that zinc supplementation may be beneficial. It can also be hypothesized that an inappropriate or inadequate Zn reserve may accelerate cell aging and potentially promote carcinogenesis due to unsatisfactory immunologic surveillance.

## Author Contributions

The author confirms being the sole contributor of this work and has approved it for publication.

## Funding

Dr. Sergi’s research has been funded by the generosity of the Stollery Children’s Hospital Foundation and supporters of the Lois Hole Hospital for Women through the Women and Children’s Health Research Institute (WCHRI, Grant ID #: 2096), Natural Science Foundation of Hubei Province for Hubei University of Technology (100-Talent Grant for Recruitment Program of Foreign Experts Total Funding: Digital PCR and NGS-based diagnosis for infection and oncology, 2017-2022), Österreichische Krebshilfe Tyrol (Krebsgesellschaft Tirol, Austrian Tyrolean Cancer Research Institute, 2007 and 2009 - “DMBTI and cholangiocellular carcinomas” and “Hsp70 and HSPBP1 in carcinomas of the pancreas”), Austrian Research Fund (Fonds zur Förderung der wissenschaftlichen Forschung, FWF, Grant ID L313-B13), Canadian Foundation for Women's Health (“Early Fetal Heart-RES0000928”), Cancer Research Society (von Willebrand factor gene expression in cancer cells), Canadian Institutes of Health Research (Omega-3 Fatty Acids for Treatment of Intestinal Failure Associated Liver Disease: A Translational Research Study, 2011-2014, CIHR 232514), and the Saudi Cultural Bureau, Ottawa, Canada. The funders had no role in study design, data collection, and analysis, decision to publish, or preparation of the manuscript.

## Conflict of Interest

The author declares that the research was conducted in the absence of any commercial or financial relationships that could be construed as a potential conflict of interest.

## Publisher’s Note

All claims expressed in this article are solely those of the authors and do not necessarily represent those of their affiliated organizations, or those of the publisher, the editors and the reviewers. Any product that may be evaluated in this article, or claim that may be made by its manufacturer, is not guaranteed or endorsed by the publisher.
